# Histone deacetylase inhibitor treatment induces ‘BRCAness’ and synergistic lethality with PARP inhibitor and cisplatin against human triple negative breast cancer cells

**DOI:** 10.18632/oncotarget.2154

**Published:** 2014-06-30

**Authors:** Kyungsoo Ha, Warren Fiskus, Dong Soon Choi, Srividya Bhaskara, Leandro Cerchietti, Santhana G. T. Devaraj, Bhavin Shah, Sunil Sharma, Jenny C. Chang, Ari M. Melnick, Scott Hiebert, Kapil N. Bhalla

**Affiliations:** ^1^ Georgia Regents University, Augusta, GA; ^2^ Houston Methodist Research Institute, Houston, TX; ^3^ Huntsman Cancer Institute, Salt Lake City, UT; ^4^ Weill Cornell Medical College, New York, NY; ^5^ Vanderbilt University, Nashville, TN

**Keywords:** HDAC inhibitor, Triple Negative Breast Cancer, PARP inhibitor, BRCAness

## Abstract

There is an unmet need to develop new, more effective and safe therapies for the aggressive forms of triple negative breast cancers (TNBCs). While up to 20% of women under 50 years of age with TNBC harbor germline mutations in BRCA1, and these tumors are sensitive to treatment with poly(ADP) ribose polymerase inhibitors, a majority of TNBCs lack BRCA1 mutations or loss of expression. Findings presented here demonstrate that by attenuating the levels of DNA damage response and homologous recombination proteins, pan-histone deacetylase inhibitor (HDI) treatment induces ‘BRCAness’ and sensitizes TNBC cells lacking BRCA1 to lethal effects of PARP inhibitor or cisplatin. Treatment with HDI also induced hyperacetylation of nuclear hsp90. Similar effects were observed following shRNA-mediated depletion of HDAC3, confirming its role as the deacetylase for nuclear HSP90. Furthermore, cotreatment with HDI and ABT-888 induced significantly more DNA strand breaks than either agent alone, and synergistically induced apoptosis of TNBC cells. Notably, co-treatment with HDI and ABT-888 significantly reduced *in vivo* tumor growth and markedly improved the survival of mice bearing TNBC cell xenografts. These findings support the rationale to interrogate the clinical activity of this novel combination against human TNBC, irrespective of its expression of mutant BRCA1.

## INTRODUCTION

DNA damage is caused by exposure of cells to a variety of agents, including environmental carcinogens, reactive oxygen species from cellular metabolism, UV, ionizing radiation, and chemotherapeutic drugs that target DNA [1, 2]. Lesser and subtle forms of DNA damage, such as oxidative lesions, alkylation of bases, DNA adducts and single strand breaks (SSBs), are repaired by the base excision repair (BER) or nucleotide excision repair (NER) mechanisms [1, 2]. More substantial and lethal DNA damage in the form of double strand breaks are repaired either by the error prone non-homologous end joining (NHEJ) through direct ligation of the DSB ends in the G0/G1 phase of the cell cycle, or by the homologous recombination (HR) mechanism, which accurately restores the genomic sequence in the cell cycle S and G2 phases by utilizing the sister chromatid as template for repair [1, 2]. HR is mediated by BRCA1, BRCA2 and RAD52 proteins while NHEJ involves KU70/80, DNA-PK and DNA ligase IV.

At the cellular level, DNA damage triggers the DNA damage response (DDR), which consists of a tightly coordinated signaling pathway, involving the sensing of DNA damage, the assembly of DNA repair factors, cell cycle transit arrest and DNA repair, all designed to maintain genome stability [1-3]. Following the recognition of DNA damage by the sensor proteins: RPA (Replication Protein A) detecting SSBs and MRN (MRE11-RAD50-NBS1) complex detecting the DSBs, the PIKK (phosphatidylinositol 3-kinase-related kinases) family members Ataxia Telangiectasia-and Rad3-related (ATR) and ATM (ataxia-telangiectasia mutated) kinase are activated [1, 4, 5]. Activated ATR assembled at the DNA lesion or stalled replication fork phosphorylates the checkpoint protein 1 (CHK1), a serine/threonine kinase, which is necessary for the activation of S and G2 cell cycle checkpoint [1, 4, 5]. This inhibits cell cycle progression, especially entry into mitosis [5]. ATR is also known to phosphorylate BRCA1 and FANCD2, thereby regulating not only cell cycle but also DNA repair [1, 5]. Thus, a well- coordinated interaction among the sensor and effector DDR and DNA repair proteins orchestrates the repair of DNA lesions [1]. Previous reports have demonstrated that BRCA1, ATR and CHK1, but not ATM, are chaperoned and stabilized by the heat shock protein (HSP) 90 [6, 7]. Consistent with this, treatment with an HSP90 inhibitor was shown to degrade and deplete expression of BRCA1, ATR and CHK1, resulting in impairment of the DDR and DNA repair, which sensitized breast cancer cells to DNA damage [6, 7]. Indeed, depletion and functional impairment of DDR and HR proteins, including BRCA1, is well documented to induce genome instability and defective DNA repair, as well as to sensitize breast cancer cells to DNA damaging agents [2, 3, 8, 9].

Poly (ADP-ribose) polymerase (PARP) family member PARP1 is a nuclear protein that also binds to DNA strand breaks and nicks [10, 11]. Following this, it is catalytically activated, mediating the synthesis of PAR polymers from NAD and causing poly(ADP-ribosylation of itself and other proteins to recruit DDR factors involved in the DNA repair [2, 10-12]. PARP1 binds to the DNA single strand breaks (SSB) during base excision repair (BER) or to DNA double strand breaks (DSB) [2, 10-12]. SSBs encountered by the replication fork generate DSBs, which require DNA repair through HR [1-3]. PARP inhibition results in HR dependency for repairing DNA DSBs [10-13]. Notably, several studies have documented that cancer cells expressing BRCA1 mutation and defective DNA damage repair through HR demonstrate synthetic lethality with PARP inhibition [2, 11, 12]. However, PARP inhibitors also trap PARP1 and PARP2 to SSB, yielding PPARP-SSB complex that exerts cytotoxicity [13]. Additionally, PARP inhibitors also stimulate NHEJ, thereby inducing genomic instability and lethality, while disabling NHEJ rescues from the lethality of PARP inhibitor in cells lacking BRCA1 [14]. Thus alternative mechanisms may contribute to the lethal activity of PARP inhibitor in cancer cells lacking BRCA1.

Primary triple negative breast cancers (TNBCs), which are defined by the lack of expression of estrogen and progesterone receptors and of HER2 gene amplification and overexpression, represent approximately 16% of all breast cancers but exhibit poor clinical outcome due to aggressive biology and lack of effective therapies [15]. As much as 20% of TNBCs in women under 50 years of age harbor germline BRCA1 mutation, and these tumors exhibit DNA repair defects and greater sensitivity to treatment with PARP inhibitors and platinum chemotherapy [2, 16-18]. Previous reports have also demonstrated that the sporadic TNBC cells exhibiting similar DNA repair defects, i.e., BRCAness, are also sensitive to PARP inhibitors, cisplatin and other DNA-damaging chemotherapy [2, 19, 20]. Here, BRCAness may be caused by DNA methylation and depletion of BRCA1 expression [21], or due to mutation or depletion of other proteins involved in HR [22]. Based on this, clinical trials of PARP inhibitor in TNBC, including olaparib and veliparib, are ongoing [23].

In a previous report, we demonstrated that treatment with a pan-HDAC inhibitor induces hyperacetylation of HSP90 and disrupts its chaperone function, destabilizing and promoting proteasomal degradation and depletion of HSP90 client proteins in breast cancer cells [24]. Based on this, in the present studies we determined whether treatment with the pan-HDAC inhibitors (HDIs) vorinostat (VS) and panobinostat (PS) induces hyperacetylation of the nuclear HSP90 and causes depletion of the client DDR and HR proteins, thereby conferring BRCAness and defective DDR and HR in the TNBC cells that lack BRCA1 mutation. Additionally, here, we determined which of the nuclear class I HDACs is mechanistically involved in de-acetylating the nuclear HSP90 [25]. Concomitantly, we also determined whether HDI mediated defective DDR and HR would sensitize TNBC cells to the *in vitro* and *in vivo* activity of PARP inhibitor. This approach was further prompted by the previous observations that treatment with HDI induces ROS and DNA damage, as well as lowers the threshold for apoptosis by inducing the pro-death members of the BCL2 family, e.g. BAX and BIM, while simultaneously attenuating the pro-survival proteins e.g. BCL-x_L_ and MCL-1 [25, 26]. Collectively, our findings here demonstrate that co-treatment with HDI and PARP inhibitor or cisplatin exerts synergistic lethality in TNBC cells, which is associated with increased DNA damage coupled with HDI-mediated depletion of DDR (ATR and CHK1) and HR proteins (BRCA1 and RAD52) in TNBC cells.

## RESULTS

### Treatment with panobinostat induces reactive oxygen species and inhibits activation of DNA damage responses

**Figure 1:**
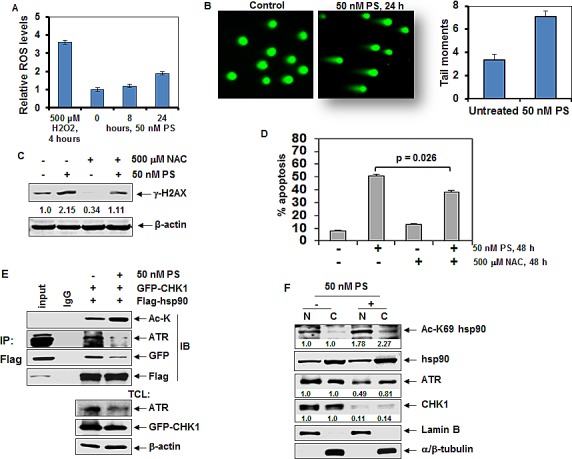
Treatment with PS induces hyperacetylation of nuclear hsp90, disrupts chaperone interaction of hsp90 with ATR and CHK1 and induces DNA damage and apoptosis of cancer cells A. MCF7 cells were plated in 96 well plates and incubated overnight at 37°C. The next day, cells were treated with 50 nM of PS for 8 to 24 hours. At the end of treatment, the relative reactive oxygen species (ROS) were measured using a microplate reader. As a positive control, cells were treated with 500 μM H_2_O_2_ for 4 hours. Post-treatment ROS levels were compared to control ROS levels and values represent the mean ± S.E.M from three independent experiments. B. MCF7 cells were treated with 50 nM PS for 24 hours. At the end of treatment, cells were analyzed by neutral comet assay. C. Immunoblot analyses of γ-H2AX and β-actin in the cell lysates from MCF7 cells treated with 50 nM PS and/or 500 μM N-acetyl cysteine (NAC) for 8 hours. D. MCF7 cells were treated with 50 nM PS and/or 500 μM N-acetyl cysteine (NAC) as indicated. Following treatment, the % annexin V-positive apoptotic cells was determined by flow cytometry. E. HeLa cells were cotransfected with FLAG-tagged hsp90 (F-hsp90) and GFP-tagged CHK1 (GFP-CHK1) constructs for 24 hours. Following this, cells were treated with 50 nM PS for 24 hours. Cell lysates were prepared and FLAG-hsp90 was immunoprecipitated using anti-FLAG (M2) antibody. Immunoblot analyses were performed for acetyl-lysine (Ac-K), ATR, GFP or FLAG. Alternatively, immunoblot analyses were performed for ATR, GFP-CHK1 and β-actin on the total cell lysates. F. HeLa cells were treated with 50 nM PS for 24 hours. At the end of treatment, nuclear and cytoplasmic fractions were prepared and immunoblot analyses were performed for acetyl lysine (K) 69 hsp90 (Ac-K69 hsp90), ATR, CHK1, and hsp90. The expression levels of lamin B and α/β-tubulin served as the fraction and loading controls.

Previous reports have shown that HDAC inhibitor-induced cell death is associated with production of reactive oxygen species (ROS) [27]. We first determined the effects of treatment with the pan-histone deacetylase inhibitor, panobinostat (PS) on induction of ROS in breast cancer cells. Figure 1A shows that treatment with PS dose-and time-dependently induced ROS (~2 fold induction with 50 nM of PS) in the MCF7 cells. HDAC inhibitor-mediated induction of ROS was associated with DNA damage and DNA double strand breaks, as shown by the increased tail moments determined by the neutral comet assay as well as by increase in the γ-H2AX levels (Figure 1B and 1C). We next evaluated whether PS-induced ROS was mechanistically linked to PS mediated DNA damage. As shown in Figure 1C and 1D, co-treatment with the free radical scavenger N-acetylcysteine (NAC) attenuated PS-mediated induction of γ-H2AX and apoptosis in MCF7 cells, indicating that ROS contributes to PS-induced DNA damage (p=0.026).

### Treatment with PS induces hyperacetylation of nuclear and cytoplasmic hsp90 and inhibits the chaperone association of ATR and CHK1 with hsp90

We had previously demonstrated that treatment with PS induces hyperacetylation of hsp90, thereby inhibiting its chaperone association with its client proteins [24]. Further, treatment with the hsp90 inhibitor AUY922 was also demonstrated to disrupt the chaperone association of hsp90 with ATR and CHK1, thereby depleting their expression levels in breast cancer cells [6]. Collectively based on these findings, we next determined the effects of PS on the chaperone association of ATR and CHK1 with hsp90. Figure 1E shows that in HeLa cells with ectopic expression of FLAG-tagged hsp90 (FLAG-hsp90) and GFP-tagged CHK1 (GFP-CHK1), treatment with PS induced hyperacetylation of FLAG-hsp90 and inhibited the binding of ATR and GFP-CHK1 to hsp90. We next determined the effects of PS treatment on the acetylation of hsp90 and expression levels of ATR and CHK1 in the nucleus versus the cytoplasm. As shown in Figure 1F, treatment with 50 nM of PS markedly induced acetylation of hsp90 in the nuclear and cytosolic fractions, which was associated with depletion of CHK1 more than ATR expression in the nucleus and the cytosolic fraction of HeLa cells. In contrast, the expression levels of the total hsp90, and the levels of the control proteins Lamin B (nucleus) and α-tubulin (cytosol) were unaffected.

### Treatment with panobinostat or vorinostat depletes BRCA1, ATR and CHK1 expression levels and induces apoptosis of TNBC cells

**Figure 2:**
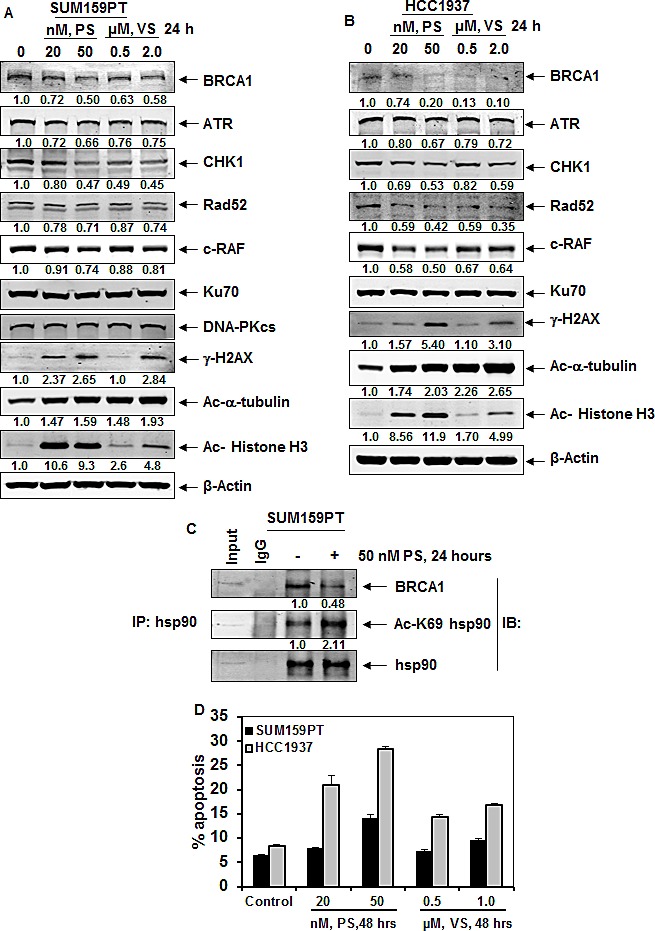
Treatment with HDAC inhibitors disrupt chaperone association of hsp90 with BRCA1, deplete BRCA1, ATR and CHK1 expression levels, as well as induce DNA damage and apoptosis of breast cancer cells A-B. SUM159PT and HCC1937 cells were treated with the indicated concentrations of panobinostat (PS) or vorinostat (VS) for 24 hours and immunoblot analyses were performed as indicated. The expression levels of β-actin in the lysates served as the loading control. Numbers beneath the bands represent densitometry analysis performed on representative blots and are relative to the untreated control cells. C. SUM159PT cells were treated with 50 nM of PS as indicated. Cell lysates were prepared and hsp90 was immunoprecipitated. Immunoblot analyses were performed for BRCA1 and acetylated hsp90. The blot was stripped and re-probed for total hsp90 expression. D. Percent apoptosis of SUM159PT and HCC1937 induced by treatment with the indicated concentrations of PS or VS for 48 hours. Columns, mean of three independent experiments; Bars, standard error of the mean.

**Figure 3:**
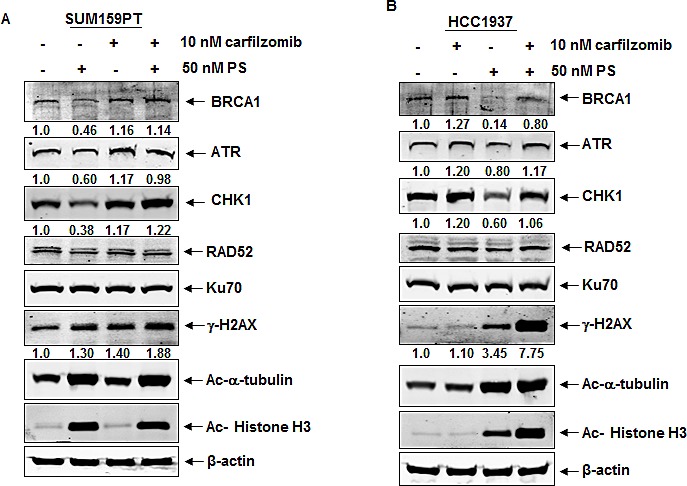
Treatment with panobinostat induces proteasomal degradation of BRCA1, ATR and CHK1 in breast cancer cells A-B. SUM159PT and HCC1937 cells were treated with 50 nM PS and/or 10 nM carfilzomib (CZ), as indicated, for 24 hours. Following this, cell lysates were prepared and immunoblot analyses were performed for the expression levels of BRCA1, ATR, CHK1, RAD52, KU70, γ-H2AX, acetylated α-tubulin, acetylated histone H3 and β-actin in the cell lysates. The numbers underneath the bands represent densitometry relative to the untreated cells.

We next determined the effects of treatment with the PS or VS on the expression levels of the DNA damage response and on the DNA repair proteins in the triple negative breast cancer cells lines SUM159PT (BRCA1 wild-type) and HCC1937 (BRCA1 mutant). As shown in Figure 2A and 2B, treatment with clinically achievable, biologically active concentrations of PS and VS depleted the expression levels of ATR and CHK1, as well as, of the HR proteins BRCA1 and RAD52 in the two cell lines. Notably, while treatment with PS attenuated mutant BRCA1 similar to un-mutated BRCA1, PS had no effect on the levels of the NHEJ proteins KU70 and DNA-PKcs. As previously reported, treatment with the pan-HDAC inhibitor VS or PS concomitantly increased the levels of γ-H2AX and induced the acetylation of histone H3 and α-tubulin [28], while simultaneously depleting the levels of c-RAF in SUM159PT and HCC1937 cells. Figure 2C demonstrates that treatment with PS also induced the hyperacetylation of hsp90, and concomitantly inhibited the chaperone association of hsp90 with its client proteins BRCA1 in SUM159PT cells (Figure 2C). Treatment with VS or PS dose-dependently induced apoptosis in HCC1937 and SUM159PT cells, although HCC1937 cells were more sensitive to the lethal effects of PS (Figure 2D). We next confirmed that, following HDAC inhibitor treatment, the reduced chaperone association with hsp90 leads to proteasomal degradation and depletion of the client proteins. As shown in Figure 3A and 3B, co-treatment with the proteasome inhibitor carfilzomib (CZ), restored the levels of PS-mediated depletion of the levels of the hsp90 client proteins, i.e., ATR, CHK1 and BRCA1. Co-treatment with CZ also partially restored RAD52 expression levels. While CZ treatment alone had no effect on the levels of Ku70, acetylated α-tubulin or acetylated histone H3, co-treatment with CZ augmented PS-induced γ-H2AX and acetylated histone H3 levels in HCC1937 more so than in SUM159T cells (Figure 3A and 3B).

### Depletion of HDAC3 induces hyperacetylation of nuclear hsp90 that leads to depletion of its client DDR and HR proteins

**Figure 4:**
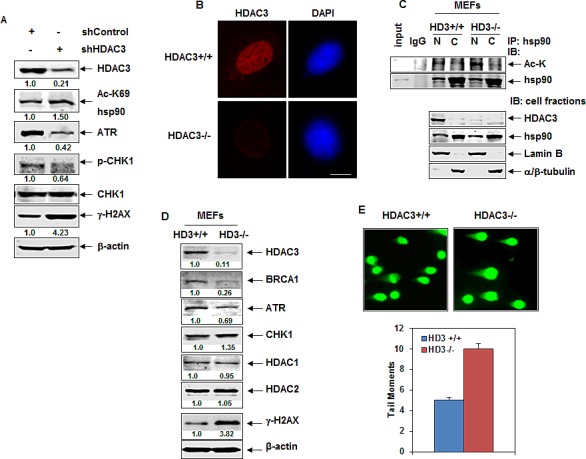
Knockdown of HDAC3 by shRNA or genetic deletion of Hdac3 induces hyperacetylation of nuclear hsp90 and attenuates the expression of ATR A. HeLa cells were transiently transfected with control or HDAC3 shRNA constructs and incubated for 96 hours. Then, immunoblot analyses were performed for the expression levels of HDAC3, ATR, p-CHK1, CHK1, γ-H2AX and -actin in the lysates. B. *Hdac3^FL/+^-Cre-ER^+^* and *Hdac3^FL/−^-Cre-ER^+^* MEFs were plated on a chamber slide and incubated overnight at 37°C. The next day, cells were treated with 1 μM tamoxifen for 72 hours. Following this, cells were stained with anti-HDAC3 antibodies and imaged by confocal immunoflourescent microscopy. The scale bar represents 10 μm. C. Hdac3^FL/+^-Cre-ER+ and Hdac3^FL/−^-Cre-ER+ MEFs were treated with 1 μM tamoxifen for 72 hours. Following this, nuclear and cytoplasmic fractions were prepared and hsp90 was immunoprecipitated. Immunoblot analyses were performed for acetyl-lysine (Ac-K) and hsp90. Alternatively, immunoblot analyses were performed for HDAC3 and hsp90 on the cellular fractions. The expression levels of lamin B and α/β-tubulin served as the fraction and loading control. D. *Hdac3^FL/+^-Cre-ER+* and *Hdac3^FL/−^-Cre-ER+* MEFs were treated with 1 μM tamoxifen for 72 hours. Then, immunoblot analyses were performed for the expression levels of HDAC3, BRCA1, ATR, CHK1, HDAC1, HDAC2, γ-H2AX and β-actin in the cell lysates E. Hdac3^FL/+^-Cre-ER+ and Hdac3^FL/−^-Cre-ER+ MEFs were treated with 1 M tamoxifen for 72 hours. Then, cells were analyzed by neutral comet assay. (upper panel) Representative images from three independent experiments are shown. (lower panel) The mean number of tail moments for 100 cells of each condition.

We had previously demonstrated that the class IIB HDAC6, which is predominantly in the cytosol, is the deacetylase for the predominantly cytosolic hsp90. Therefore, we next determined which among the class I HDACs is responsible for de-acetylating the smaller nuclear fraction of hsp90, such that its inhibition by PS or VS would lead to hyperacetylation of the nuclear hsp90, resulting in destabilization of the chaperone association of hsp90 with the DDR and HR proteins in the nucleus. Therefore, we determined the effects of HDAC 1, 2, or 3 knockdown by shRNA on the acetylation status of nuclear hsp90. In HeLa cells, following transient transfection, the shRNA to HDAC1, HDAC2, or HDAC3 depleted the mRNA, as well as attenuated the proteins levels of HDAC1, HDAC2 or HDAC3 in the nucleus not in the cytoplasm, respectively (Supplemental Figure 1A, 1B and 1C). Notably, it was only the depletion of HDAC3 in the nucleus that induced the hyperacetylation of nuclear hsp90, demonstrated in the hsp90 immunoprecipitates from the nuclear fraction followed by immunoblot analyses with the anti-acetyl lysine antibody, or by the immunoblot analyses of the nuclear extracts utilizing the acetylated K69-hsp90 antibody. (Supplemental Figure 1B). Importantly, the shRNA-mediated depletion of HDAC3 reduced the protein levels of ATR but not of ATRIP, which is a co-activator of ATR (Figure 4A and Supplemental Figure 1C) [1, 4, 5]. The shRNA-mediated depletion of HDAC3 also reduced the levels of p-ATR and p-CHK1 in HeLa cells, indicating that HDAC3 depletion inhibits the ATR-CHK1 pathway in cancer cells (Figure 4A). As previously observed with HDAC inhibitor treatment, shRNA-mediated depletion of HDAC3 caused a significant (approximately 4-fold) up-regulation of the DNA damage marker γ-H2AX in cancer cells (Figure 4A). Unlike the effect of PS treatment, shRNA mediated knockdown of HDAC3 alone increased the levels of CHK1 in HeLa cells (Supplemental Figure 1C).

### Genetic knockdown of Hdac3 results in depletion of the levels of DDR and HR proteins

Next, utilizing the Hdac3 knockout mouse embryonic fibroblasts (***Hdac3^FL/+^-Cre-ER^+^*** and ***Hdac3^FL/−^-Cre-ER^+^*** MEFs) [29, 30], we further confirmed the effects of genetic knockdown of HDAC3 on the acetylation of nuclear hsp90 and on the expression of the DDR and HR proteins. As shown in Figure 4B, compared to the Hdac3^+/+^ MEFs, Hdac3^−/−^ MEF cells exhibited absence of HDAC3, as determined by immunofluorescence microscopy following staining with anti-HDAC3 antibody. Additionally, in the hsp90 immunoprecipitates from the nuclear but not the cytoplasmic fractions of Hdac3^-/-^ MEFs, higher levels of hyperacetylated hsp90 were noted; the levels of lamin B and tubulin served as the controls for the purity of the fractions (Figure 4C). Similar to cancer cells in which HDAC3 was depleted by shRNA, Hdac3-deficient MEFs also exhibited decreased expression levels of BRCA1 and ATR but increased levels of CHK1 and γ-H2AX (Figure 4D). In addition, compared to control MEFs, Hdac3^−/−^ MEFs exhibited 2-fold greater tail moments as measured by the comet assay, indicating that deletion of Hdac3 results in increased levels of DNA damage in the MEFs (Figure 4E).

### Co-treatment with pan-HDAC inhibitor significantly enhances PARP inhibitor-mediated DNA damage and synergistically induces apoptosis of TNBC cells

**Figure 5:**
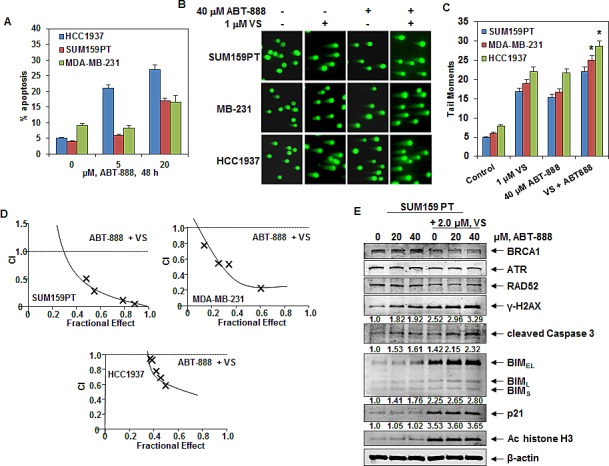
Co-treatment with HDAC inhibitor significantly enhances ABT-888-mediated DNA damage and synergistically induces apoptosis of breast cancer cells A. HCC1937 and SUM159PT cells were treated with ABT-888 for 48 hours and the % of apoptotic cells were determined by flow cytometry. B. SUM159PT, MDA-MB-231 and HCC1937 cells were treated with ABT-888 and/or 1 μM of VS for 24 hours, then cells were analyzed by neutral comet assay. Representative images from 3 independent experiments are shown. C. Cells were treated as in (B). The graph shows the mean tail moments for 100 cells for each condition in each cell line. * indicates significantly greater tail moments in MDA-MB-231 and HCC1937 treated with the ABT-888 and VS, compared to treatment with either agent alone (p< 0.05) D. SUM159PT, HCC1937 and MDA-MB-231 cells were treated with ABT-888 and PS for 48 hours and the % apoptotic cells was determined by flow cytometry. Median dose effect and isobologram analyses were performed using Calcusyn. Combination index (CI) values less than 1.0 indicate a synergistic interaction of the two agents in the combination. E. Immunoblot analyses of SUM159PT cells following treatment with the indicated concentrations of ABT-888 and/or VS for 24 hours. The expression levels of β-actin in the lysates served as the loading control. Numbers beneath the bands represent densitometry analysis performed on representative blots and are relative to the untreated control cells.

Previous studies have demonstrated that TNBC cells, especially those with BRCA1 mutation are sensitive to treatment with PARP inhibitors [2, 17, 18]. Consistent with these reports, treatment with the PARP inhibitor ABT-888 induced significantly more apoptosis in BRCA1-mutant HCC1937 cells, as compared to those TNBC cell types lacking BRCA1 mutation (Figure 5A). We next asked whether co-treatment with a pan-HDAC inhibitor, which causes depletion of DDR and HR proteins, would further enhance the DNA damaging effects of ABT-888. As shown in Figure 5B, compared to treatment with either agent alone, co-treatment with VS and ABT-888, significantly enhanced ABT-888-mediated DNA damage in the TNBC cells resulting in increased comet tail moments (p<0.05). Figure 5C demonstrates that BRCA1-mutant HCC1937 cells were the most sensitive to the combined effects of VS and ABT-888. Consistent with this, co-treatment with VS and ABT-888 synergistically induced apoptosis of the TNBC cells as determined by median dose effect and isobologram analyses (Figure 5D and Supplemental Figure 2). Combination indices (CI) were calculated for the combinations in each cell line. All CI values were less than 1.0 indicating a synergistic interaction between ABT-888 and VS. In contrast, treatment with ABT-888 and/or VS induced relatively less apoptosis in the normal CD34+ bone marrow progenitor cells, with less than 20% cell death induced by the combination (Supplemental Figure 3). While depleting BRCA1, RAD52 and ATR the synergistic activity of co-treatment with VS and ABT-888 was associated with greater induction of γ-H2AX, p21, cleaved caspase 3 and the isoforms of BIM in the SUM159PT cells (Figure 5E).

### Co-treatment with ABT-888 and VS causes tumor growth delay and significantly improves survival of nude mice bearing MDA-MB-231 xenografts

**Figure 6:**
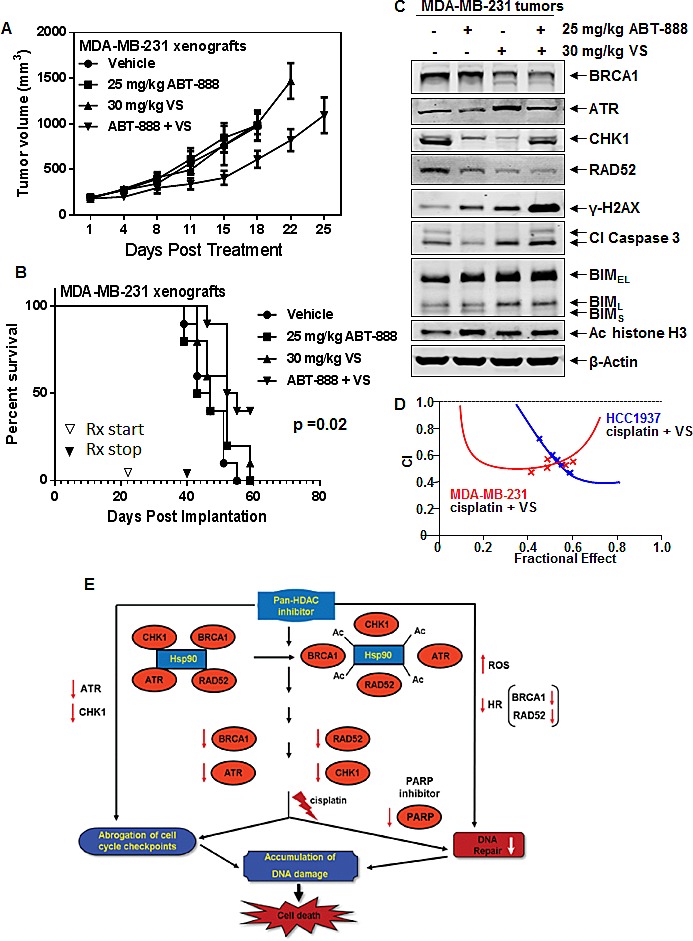
Co-treatment with VS and ABT-888 significantly inhibits tumor growth and improves the survival of NOD/SCID mice bearing MDA-MB-231 xenografts A. Mean tumor volume of mice treated with vehicle, ABT-888 and/or VS for 3 weeks. Mice treated with ABT-888 and VS displayed significantly smaller tumors than mice treated with ABT-888 alone (p=0.037) or VS alone (p=0.04). B. Kaplan-Meier survival plot of the mice treated with vehicle, ABT-888, VS, or ABT-888+VS. Mice treated with the combination of ABT-888 and VS demonstrated significantly improved survival (p=0.02) by Log rank (Mantel-Cox) test. C. Representative immunoblots of BRCA1, ATR, CHK1, RAD52, γ-H2AX, cleaved Caspase 3, acetyl histone H3, BIM and β-actin in cell lysates from tumors harvested from mice following 1 week of treatment with ABT-888 and/or VS. D. HCC1937, MDA-MB-231 and SUM159PT cells were treated with cisplatin and vorinostat (VS) for 48 hours and the % apoptotic cells was determined by flow cytometry. Median dose effect and isobologram analyses were performed using Calcusyn. Combination index (CI) values less than 1.0 indicate a synergistic interaction of the two agents in the combination. E. Pan-HDAC inhibitor, by inhibiting HDAC6 levels and activity, induces acetylation and inhibits the chaperone activity of hsp90. This disrupts the chaperone association of hsp90 with its client proteins, such as ATR, BRCA1, RAD52 and CHK1, leading to depletion of their expression levels. Cisplatin treatment leads to decreases in DNA repair and abrogation of cell cycle checkpoints. Treatment with PARP inhibitor inhibits DNA repair leading to accumulation of DNA damage. Combined treatment with HDAC inhibitor and PARP inhibitor leads to greater DNA damage and increased cell death through increased ROS and inhibition of homologous recombination due to depletion of BRCA1 and RAD52. Combined treatment with pan HDAC inhibitor and cisplatin causes greater abrogation of cell cycle checkpoints through HDAC inhibitor-mediated depletion of ATR and CHK1. In addition, accumulation of DNA damage from the combined action of HDAC inhibitor and cisplatin leads to increased cell death of breast cancer cells.

We next determined the effects of treatment with ABT-888 (25 mg/kg daily by oral gavage, 5 days per week for 3 weeks) and/or VS (30 mg/kg daily, intra-peritoneal injection, 5 days per week for 3 weeks) on the tumor growth of MDA-MB-231 implanted into the mammary fat pad of female nude mice. Figure 6A shows that although mice treated with ABT-888 or VS alone had little impact on tumor growth, mice treated with the relatively short course of ABT-888 and VS exhibited significant tumor growth delay (p=0.037, for the combination vs ABT-888 alone; p=0.04, for the combination vs VS alone). As compared to the untreated control or treatment with each agent alone, co-treatment with ABT-888 and VS also significantly improved the survival of mice, as demonstrated in the Kaplan-Meier plot (p=0.02) (Figure 6B). The dose and schedule of ABT-888 and PARP, as used here for 3 weeks, did not induce any discernible toxicity in the mice. In separate cohorts of mice, we also excised the tumors following treatment for 1 week with ABT-888 and/or VS, and performed immunoblot analyses on the cell lysates. Figure 6C demonstrates that tumors from the mice treated with the combination exhibited depletion of BRCA1, RAD52, CHK1 and ATR, while simultaneously showing induction of γ-H2AX, cleaved caspase 3, and BIM (Figure 6C). Increased levels of hyperacetylated histone H3 were also noted, indicating that biologically effective levels of VS were achieved (Figure 6C).

### Co-treatment with VS synergistically enhances the activity of cisplatin in TNBC cells

Previous studies have documented the activity of cisplatin against TNBC cells, especially those expressing mutant BRCA1, which is associated with the formation of DNA adducts/crosslinks and DNA strand breaks [18, 31]. Consistent with this, we determined that treatment with cisplatin dose-dependently induced more apoptosis of HCC1937, as compared to MDA-MB-231 cells (Supplemental Figure 4A). Cisplatin also dose-dependently induced apoptosis of SUM159PT cells (data not shown). We next determined whether co-treatment with pan-HDAC inhibitor would further sensitize TNBC cells to cisplatin-induced apoptosis. Figure 6D and Supplemental Figure 4B-C demonstrate that co-treatment with VS and cisplatin synergistically induced apoptosis of HCC1937, MB-231 and SUM159PT cells, with CI values less than 1.0 in all combinations tested. Figure 6E graphically depicts the potential basis for the synergistic anti-TNBC activity of the combination of pan-HDAC inhibitor and PARP inhibitor or cisplatin. As shown, treatment with pan-HDAC inhibitor not only induces ROS and DNA damage but, by also depleting DDR (ATR and CHK1) and HR proteins (BRCA1 and RAD52), it creates ‘BRCAness’, which sensitizes TNBC cells to PARP inhibitor or DNA damage induced by cisplatin.

## DISCUSSION

Pre-clinical reports and clinical trials have recently documented the increased sensitivity of cancers expressing BRCA1 mutation to PARP inhibitor, such as veliparib, and to DNA damaging agents [2, 17, 18]. In TNBC cells expressing BRCA1 mutation and exhibiting impaired HR, the superior activity of PARP inhibitor is attributed not only to the concomitant inhibition of BER, but also to the entrapment of PARP1 and 2 and to the increased dependency on NHEJ [2, 12-14]. Consistent with this, findings presented here go further in demonstrating for the first time that HDI-mediated depletion of HR (BRCA1 and RAD52) and DDR proteins (ATR and CHK1) sensitizes TNBC cells, whether they have, or lack, BRCA1 mutation, to the PARP inhibitor veliparib and the DNA damaging agent cisplain. However, the combination of an HDI and veliparib or cisplatin was more effective against TNBC cells expressing BRCA1 mutation. These findings are also consistent with the previous reports, and highlight the underlying mechanism that down regulation of BRCA1 and RAD52 levels would confer BRCAness and undermine HR in TNBC cells lacking BRCA1 mutation [1, 2, 19]. As compared to treatment with the vehicle control or each agent alone, co-treatment with VS and ABT-888 also exerted significantly superior anti-tumor effects and improved the survival of the mice engrafted with TNBC cells. The improvement in survival was associated with a marked in vivo depletion in ATR, CHK1 and RAD52, but induction of γ-H2AX, cleaved Caspase 3 and BIM in the engrafted tumor cells following 1 week of treatment with the combination. These findings suggest that the superior anti-tumor activity of the combination is potentially due to the perturbations in the expression of these proteins.

Our findings here also illuminate the mechanism by which HDI treatment depletes the nuclear DDR and HR proteins. Treatment with the HDIs PS and VS induces hyperacetylation and inhibition of the chaperone association of nuclear HSP90 with the DDR and HR proteins. The class I HDAC3 was specifically involved in deacetylating HSP90, since its inhibition with PS or VS [32], or the genetic knockdown of HDAC3, was associated with the induction of nuclear HSP90 acetylation and depletion of the DDR and HR proteins. HDIs are also known to induce ROS and inflict DNA damage, which increases the dependency on DDR and DNA repair mechanisms for cell survival following exposure to the HDI [27, 33, 34]. Several class I and II HDACs have also been shown to promote DNA DSB repair both by HR and NHEJ [8, 35, 36]. Therefore, HDI-induced DNA damage coupled with the attenuation of the DDR and HR proteins that leads to inhibition of DNA DSB repair, in essence creates ‘double jeopardy’ for the TNBC cell survival. HDAC3 in a complex with NCOR1 and NCOR2 (SMRT) has also been shown to directly promote DNA repair and genomic stability [29, 30, 36]. In Hdac3^−/−^ cells, increased DNA damage and defective DNA DSB repair was reported [29, 30]. Consistent with this, in the present studies, inhibition of HDAC3 by treatment with HDI could also directly undermine DNA DSB repair, in addition to inducing ‘BRCAness’ through depletion of the HR proteins. This is supported by our observation that HDI treatment or genetic knockdown of HDAC3 was associated with in vitro and in vivo induction of the phosphorylation of histone H2AX on Ser139 and apoptosis of TNBC cells (Figure 5E and Figure 6C). Furthermore, the synergistic lethality of the co-treatment with HDI and veliparib was coupled with greater induction of γ-H2AX, all three isoforms of BIM and cleaved Caspase 3 in TNBC cells.

Recent studies have shown that in cells lacking BRCA1 mutation, sensitization to treatment with PARP inhibitor can also be achieved in sporadic breast cancers through depletion of BRCA1 due to promoter methylation [21]. ‘BRCAness’ and PARP inhibitor sensitivity has also been observed in cells exhibiting promoter methylation of the of the Fanconi Anemia FANCF gene [37]. Depletion and inhibition of the activity of CDK1, which phosphorylates BRCA1 and is necessary for the formation of BRCA1 foci and DNA damage repair by HR, was also reported to sensitize cancer but not the untransformed cells to PARP inhibition [38]. PTEN mutations and loss of function was also shown to be associated with defective HR and sensitization to treatment with PARP inhibitor [2, 39]. Collectively, these reports highlight that depletion and inhibition of DDR and HR proteins, as was also observed here due to treatment with HDI, lead to enhanced sensitivity to PARP inhibitor in sporadic cancers. Recently, co-treatment with PI3K inhibitor was shown to significantly enhance the activity of PARP inhibitor against BRCA-related breast cancer [40]. This would also explain why treatment with HDI, which is well documented to deplete p-AKT and p-ERK1/2 [25, 26, 41], synergistically enhanced here the activity of the PARP inhibitor veliparib against TNBC cells. Recently, mechanisms that confer resistance to PARP inhibition have also been identified in tumors with defective HR due to BRCA1 mutation. Loss of 53BP1 was demonstrated to rescue HR and confer resistance to a PARP inhibitor in cells expressing mutant BRCA1 [42, 43]. Activation of the P-glycoprotein drug efflux transporter was also shown to confer resistance to the PARP inhibitor olaparib in a BRCA1-deficient mouse mammary tumor [43]. Additionally, it is conceivable that reversion mutations that restore the open reading frame of BRCA1, as has been documented for BRCA2, may also confer resistance to PARP inhibitor in breast and ovarian cancers expressing BRCA1 mutation [2, 8, 44]. It is tempting to speculate that co-treatment with HDI, which inhibits DDR, and by attenuating the protein levels of BRCA1 and RAD52 inhibits HR, may be effective in preventing resistance to PARP inhibitor therapy. This may occur through abrogation of the secondary BRCA1 mutations that partially restore BRCA1 function, or by inhibiting HR that would blunt any restorative effects of 53BP1 loss on HR [2, 42-44]. Recently, loss of BRCA1 function was shown to be associated with the expansion of breast cancer stem and progenitor cells, conferring a dependency on the expression of the polycomb repressor 2 complex protein EZH2 [45, 46]. Additionally, HDAC3 was reported to be essential for stem/progenitor cell function and DNA replication [47]. This would suggest that co-treatment with an HDI, which has been previously demonstrated to attenuate EZH2 levels and inhibit HDAC3 [37, 48], could potentiate PARP inhibitor activity against TNBC stem/progenitor cells. Collectively, for all of the supportive rationale cited above, the findings presented here make a strong case for further testing of the combination of treatment with an HDI with PARP inhibitor and/or cisplatin against in vivo models of TNBC cells.

## MATERIALS AND METHODS

### Cell culture

The human breast cancer cell lines MCF7, MDA-MB-231 and HCC1937 and HeLa cells were obtained from American Type Culture Collection (Manassas, VA). SUM 159PT cells were obtained from Asterand (Detroit, MI). Cells were thawed and cultured for 3-5 passages, then frozen in aliquots in liquid nitrogen. All experiments with cell lines were performed within 6 months after thawing or obtaining from ATCC or Asterand. Cell line characterization was performed by ATCC or Asterand utilizing short tandem repeat (STR) profiling. HeLa cells were maintained in DMEM medium containing 10% fetal bovine serum, 1% non-essential amino acids (NEAA), and 1% penicillin/streptomycin as previously described [6, 41]. MDA-MB-231 and HCC1937 cells were cultured in RPMI160 media containing 10% FBS and 1% penicillin/streptomycin. SUM-159PT cells were cultured in Ham's F-12 with 5% Fetal Bovine Serum, insulin and hydrocortisone. All cell lines were passaged 2-3 times per week. Hdac3^FL/+^/Cre-ER^+^ and Hdac3^FL/−^/Cre-ER^+^ mouse embryonic fibroblast (MEFs) (kindly provided and characterized by Dr. Scott Hiebert) were cultured in DMEM containing 10% FBS, 1% NEAA and 0.5% penicillin/streptomycin solution. For conditional HDAC3 knockout, MEFs were treated with 1 μM tamoxifen (Sigma-Aldrich, St. Louis, MO) for 72 hours and fresh tamoxifen-containing media was added to the cells as previously described [29, 30]. Logarithmically growing cells were used for all experiments detailed below. Cells were washed free of the drugs prior to harvesting for experimentation.

### Reagents and antibodies

Carfilzomib was obtained from Selleck Chemicals. Pan-histone deacetylase (HDAC) inhibitors panobinostat (PS) and vorinostat (VS) were obtained from Novartis Pharmaceuticals (East Hanover, NJ) and Selleck Chemicals, respectively. All drugs were prepared as 10 mM stocks in 100% DMSO and stored in small aliquots at −80°C to prevent multiple free thaw cycles. Anti-phosphorylated (p)-ATR (S428), anti-p-CHK1(S345), anti-α/β tubulin, anti-DNA-PKcs, anti-acetyl histone H3 (K9/K14), anti-histone H3, anti-RAD52, anti-BRCA2 and anti-acetyl lysine antibodies were purchased from Cell Signaling Technology (Berverly, MA). Anti-hsp90α and anti-hsp70 antibody was purchased from Enzo Biosciences (Plymouth Meeting, PA). Anti-BRCA1 and anti-γ-H2AX antibodies were obtained from Millipore (Billerica, MA). Anti-CHK1, anti-ATR, anti-HDAC1, anti-HDAC2, and anti-HDAC3 antibodies were purchased from Santa Cruz Biotechnology (Santa Cruz, CA). Anti-β-actin, anti-FLAG, anti-acetylated α-tubulin and anti-GFP antibodies and short hairpin RNAs against HDAC1, HDAC2 and HDAC3 were purchased from Sigma-Aldrich (St. Louis, MO). Acetylated-K69 hsp90 (Ac-K69 hsp90) antibody was previously described [24]. Anti-c-RAF antibody was purchased from BD Transduction Labs (San Jose, CA).

### RNA interference and transfection of cDNAs

For short hairpin RNA (shRNA)-mediated down-regulation of HDAC1, 2 and 3, cells were transiently transfected with shRNAs utilizing Lipofectamine 2000 (Invitrogen, Carlsbad, CA) as previously described [49]. After 24 hours, the cells were washed with media and incubated an additional 24 to 72 hours. Then, cells were harvested for Western blot or mRNA analyses. For the ectopic over-expression of FLAG-tagged hsp90 (F-hsp90), or green fluorescent protein-tagged CHK1 (GFP-CHK1), cells were transiently transfected with plasmid vectors expressing F-hsp90, or GFP-CHK1 cDNA utilizing Lipofectamine 2000 for 48 hours. Following this, the cells were treated with panobinostat for 24 hours, and harvested for Western blot or immunoprecipitation analyses.

### Assessment of apoptosis by annexin-V staining

Untreated or drug-treated cells were stained with Annexin-V (Pharmingen, San Diego, CA) and TO-PRO-3 iodide and the percentages of apoptotic cells were determined by flow cytometry as previously described [50]. To analyze synergism between ABT-888 (ABT-888) or cisplatin and vorinostat, cells were treated with ABT-888 (10-20 μM) or cisplatin (2-10 μM) and vorinostat (0.1-2.0 μM) for 48 hours and the percentages of annexin V-positive, apoptotic cells were determined by flow cytometry. The combination index (CI) for each drug combination was calculated by median dose effect analyses (assuming mutual exclusivity) utilizing the commercially available software Calcusyn (Biosoft, Ferguson, MO). CI values of less than 1.0 represent a synergistic interaction of the two drugs in the combination.

### Measurement of intracellular reactive oxygen species (ROS)

Intracellular ROS levels were measured by a microplate reader using the fluorescent dye 5-(and-6)-carboxy-2’,7’-difluorodihydrofluorescein diacetate (carboxy-H_2_DFFDA, Invitrogen, Carlsbad, CA). Briefly, cells were plated on a 96-well plate and incubated overnight at 37°C. Then, cells were treated with PS (20 to 50 nM) for 8 to 24 hours. As a positive control, cells were treated with 500 μM H_2_O_2_ for 4 hours. At the end of treatment, cells were washed with 1X PBS and incubated in phenol red-free medium containing 5 μM carboxy-H_2_DFFDA for 20 min at 37°C. Following this, cells were washed in 1X PBS and resuspended in PBS containing 10 mM HEPES. The fluorescence was measured at 528 nm using a microplate reader (BioTek, Winooski, VT) [27].

### Nuclear and cyto solic fraction preparation

Following drug treatments or shRNA transfection, cells were washed twice with PBS and lysed in RIPA buffer (Pierce, Rockford, IL) containing Complete EDTA-free protease inhibitor (Roche Diagnostics, Indianapolis, IN). Alternatively, cells were harvested and nuclear/cytoplasmic fractions were prepared using a NE-PER extraction kit (Pierce, Rockford, IL) according to the manufacturer's protocol [49]. Total protein in the lysates was determined utilizing a BCA protein assay kit (Pierce, Rockford, IL), according to the manufacturer's protocol.

### Immunoblot analyses

Total cell lysates or nuclear and cytosolic fractions were separated by sodium dodecyl sulfate-polyacrylamide gel electrophoresis (SDS-PAGE), and transferred to PVDF-FL membranes. Blots were incubated with primary antibody overnight at 4°C, washed 3 times with 1X PBST then incubated in IRDye 680 goat anti-mouse or IRDye 800 goat anti-rabbit secondary antibodies (LI-COR, Lincoln, NE) for 1 hour, washed 3 times in 1X PBST and scanned with an Odyssey Infrared Imaging System (LI-COR, Lincoln, NE) [51]. The expression levels of β-actin were used as the loading control for the Western blots. Immunoblot analyses were performed at least twice. Representative immunoblots were subjected to densitometry analysis. Densitometry was performed using ImageQuant 5.2 (GE Healthcare, Piscataway, NJ).

### Immunoprecipitation

Immunoprecipitation of FLAG-tagged hsp90 was performed as previously described [24]. Briefly, cell lysates were mixed with anti-FLAG (M2), anti-hsp90, anti-ATR or anti-GFP antibody, and incubated overnight at 4 °C with rotation. Protein G-agarose beads were added to the antibody/lysate mix and incubated for 3 hours. The beads were washed 3 times in lysis buffer (20 mM Tris-HCl, 150 mM NaCl and 1% Triton X-100) and sample buffer was added prior to boiling. SDS-PAGE and immunoblot analyses were performed as described above.

### RNA isolation and quantitative reverse transcription-polymerase chain reaction

Total RNA was extracted using an RNAqueous-4PCR kit (Ambion, Austin, TX) according to the manufacturer's protocol and converted into cDNA using the High Capacity cDNA Reverse Transcription Kit (Applied Biosystems, Carlsbad, CA) [49]. Primers for quantitation of ATR, CHK1, HDAC1, 2 and 3 mRNA expression were purchased from Origene (Rockville, MD). Relative mRNA expressions were normalized to glyceraldehyde-3-phosphate dehydrogenase (GAPDH).

### Immunofluorescent microscropy

MEF cells were grown on chamber slides overnight at 37°C. Following this, cells were treated with 50 nM PS for 24 hours. At the end of treatment, cells were fixed with 4% paraformaldehyde for 10 minutes and permeabilized with 0.5% Triton X-100 for 10 minutes. The slides were blocked with 3% BSA for 30 minutes and incubated with anti-hsp90α, anti-Ac-K69 hsp90α, anti-acetyl α-tubulin, anti-ATR or anti-HDAC3 antibody for 2 hours at 37°C [24, 41, 49]. The slides were washed three times in 1X PBS and incubated with Alexa Fluor 594 or Alexa Fluor 488-conjugated species-specific secondary antibodies for 1 hour. After three washes with 1X PBS, the cells were counterstained using SlowFade Gold anti-fade reagent with DAPI (Invitrogen, Carlsbad, CA) and imaged using a AxioCam MRm microscope with a 40x (0.6 Na) objective (Carl Zeiss, Heidelberg, Germany).

### Analysis of DNA damage by comet assay

For the measurement of DNA damage repair, cells were harvested after treatment of PS or VS with or without ABT-888 and neutral comet assay was performed, as previously described [6].

### *In vivo* model of breast cancer

All *in vivo* studies were approved by, and conducted in accordance with the guidelines of the IACUC at Houston Methodist Research Institute. MDA-MB-231 cells (5 million cells per mouse) were injected into the mammary fat pad of female nude mice (The Jackson Laboratory, Bar Harbor, ME). Tumor growth was monitored by external caliper measurement. General condition of the mice was monitored daily. Treatment was initiated when mean tumor volume was approximately 200 mm^3^ for all groups. Mice (n=10 per cohort) were treated with ABT-888 (25 mg/kg, diluted in sterile, acidified normal saline (pH 4.0) P.O. daily, 5 days per week), vorinostat (30 mg/kg diluted in DMSO, i.p. daily, 5 days per week) or ABT-888 and vorinostat for 3 weeks. Mice were humanely euthanized when tumor volumes exceeded 1500 mm^3^. Survival of the mice is reported by a Kaplan Meier plot [27, 33]. For biomarker analysis, a cohort of mice was treated with ABT-888 and/or VS for 1 week and then humanely euthanized. Tumors were excised and cell lysates were prepared for immunoblot analysis.

### Statistical analyses

Data are expressed as mean plus or minus standard error of the mean (SEM). Significant differences between a population of breast cancer cells treated with PS and/or NAC or VS and ABT-888 were determined using a two-tailed, paired t-test within an analysis package add-on in Microsoft Excel. *P* values less than 0.05 were assigned significance. A two-way ANOVA analysis was used to determine significant differences in mean tumor volumes in the xenograft model. *P* values less than 0.05 were assigned significance. Significant differences in survival of animals were calculated using a Log-rank (Mantel-Cox) test. *P* values less than 0.05 were assigned significance.

## SUPPLEMENTAL MATERIALAND FIGURES


