# Supplementary material for: Histone deacetylase inhibitor treatment induces ‘BRCAness’ and synergistic lethality with PARP inhibitor and cisplatin against human triple negative breast cancer cells
Source: Oncotarget. 2014 Jun 30;5(14):5637–50. doi: 10.18632/oncotarget.2154 (PMC4170637; doi:10.18632/oncotarget.2154)
Supplement: Supplementary file 1 [file oncotarget-05-5637-s001.pdf]

Histone deacetylase inhibitor treatment induces 'BRCAness' and synergistic lethality with PARP inhibitor and cisplatin against human triple negative breast cancer cells

Supplemental Material

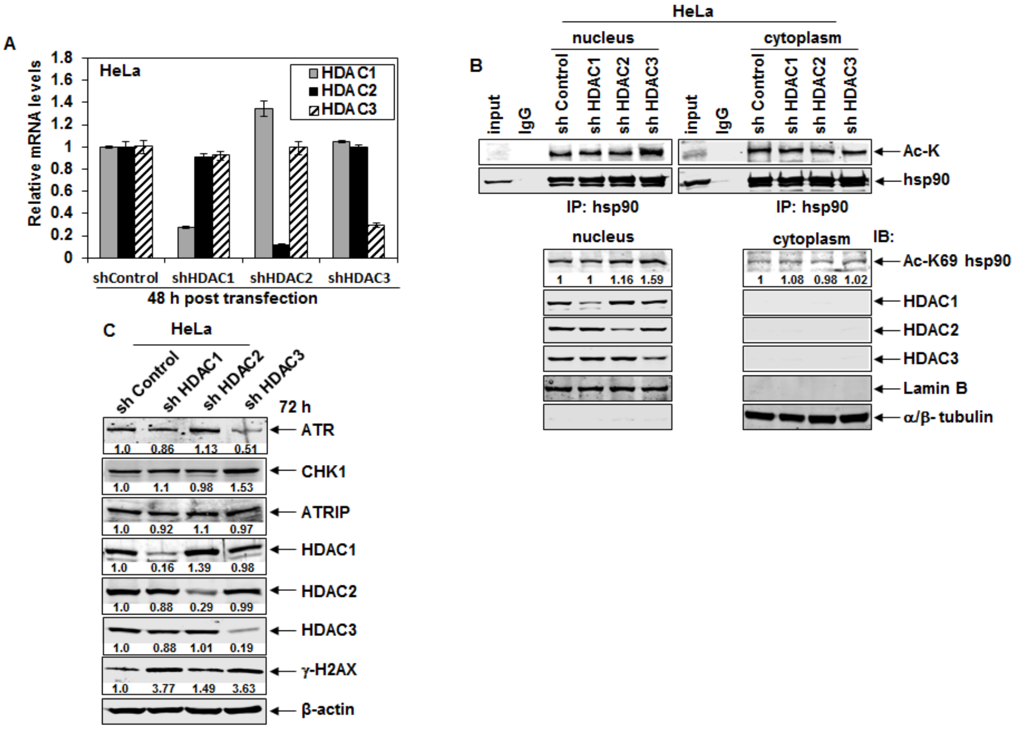

Supplemental Figure 1: Knockdown of HDAC3 induces hyperacetylation of nuclear hsp90. A. HeLa cells were transiently transfected with non-targeted control, HDAC1, 2 or 3 shRNAs and incubated for 48 hours. Following this, total RNA was isolated and quantitative RT-PCR was performed for the expression levels of HDAC1, HDAC2 and HDAC3. Relative mRNA expression was normalized against GAPDH. **B**. HeLa cells were transfected with non-targeting control, HDAC1, 2 or 3 shRNA and incubated for 72 hours. Following this, nuclear and cytoplasmic fractions were harvested and hsp90 was immunoprecipitated. Immunoblot analyses were performed for acetyl-lysine (Ac-K), lysine (K) 69-acetylated hsp90 (Ac-K69 hsp90), HDAC1, HDAC2 and HDAC3. The expression levels of lamin B and α/β-tubulin served as the loading and fraction controls. **C**. HeLa cells were transiently transfected with non-targeted control, HDAC1, 2 or 3 shRNAs and incubated for 72 hours. Following this, total cell lysates were prepared and immunoblot analyses were conducted for the expression levels of ATR, CHK1, ATRIP, HDAC1, HDAC2, HDAC3, γ-H2AX and β-actin in the cell lysates.

A

SUM159PT

ABT888 (µM)	VS (µM)	Fa	CI
12.5	1.25	0.538	0.330
15	1.5	0.473	0.528
17.5	1.75	0.777	0.137
20	2	0.872	0.075

HCC1937

ABT-888 (µM)	VS (µM)	Fa	CI
20	0.1	0.359	0.963
20	0.25	0.409	0.794
20	0.5	0.374	0.942
20	0.75	0.446	0.706
20	1	0.487	0.604

MDA-MB-231

ABT888 (µM)	VS (µM)	Fa	CI
10	0.25	0.126	0.790
10	0.5	0.244	0.562
10	0.75	0.328	0.551
10	1	0.592	0.240

Supplemental Figure 2: Co-treatment with vorinostat and ABT-888 synergistically induces apoptosis of breast cancer cells. SUM159PT, HCC1937 and MDA-MB-231 cells were treated with ABT-888 and VS for 48 hours and the % apoptotic cells were determined by flow cytometry. Median dose effect and isobologram analyses were performed (assuming mutual exclusivity) using Calcusyn. Combination index (CI) values less than 1.0 indicate a synergistic interaction of the two agents in the combination. Figure shows the doses of each agent, the fraction of cells affected by the combination and the calculated CI value for each.

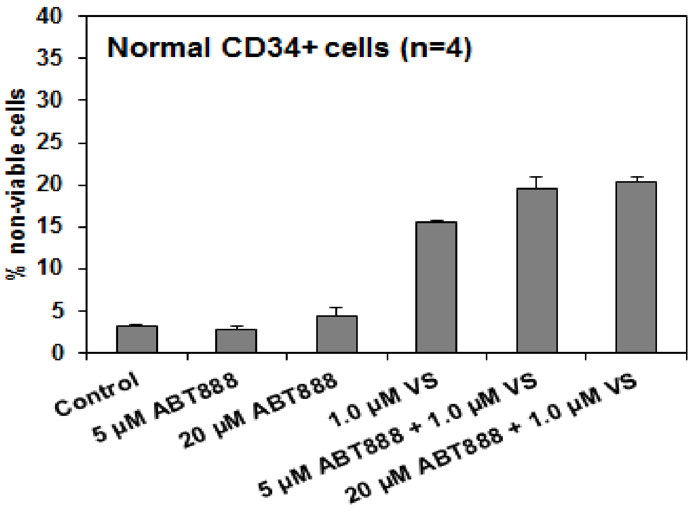

Supplemental Figure 3: Treatment with ABT-888 and VS is relatively sparing of normal CD34+ cells. Normal CD34+ cells were treated with the indicated concentrations of ABT888 and/or VS for 48 hours. At the end of treatment, cells were washed with 1X PBS then stained with propidium iodide. The % of non-viable cells was determined by flow cytometry. Columns, mean of the 4 samples; Bars, standard error of the mean.

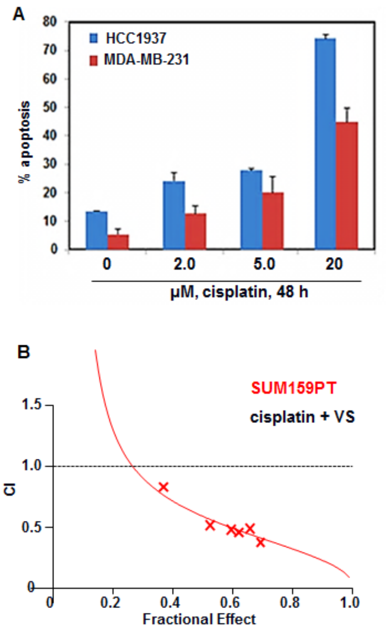

C

MDA-MB-231

VS (µM)	Cisplatin (µM)	Fa	CI
0.2	2	0.436	0.360
0.4	4	0.501	0.444
0.5	5	0.499	0.562
0.75	7.5	0.577	0.506
1.0	10	0.606	0.563

HCC1937

VS (µM)	Cisplatin (µM)	Fa	CI
0.2	10	0.454	0.722
0.3	10	0.535	0.559
0.5	10	0.514	0.598
0.6	10	0.554	0.527
0.75	10	0.591	0.468

SUM159PT

VS (µM)	Cisplatin (µM)	Fa	CI
0.75	5	0.368	0.839
0.9	6	0.523	0.524
1.12	7.46667	0.594	0.488
1.2	8	0.621	0.467
1.35	9	0.693	0.382
1.5	10	0.657	0.500

Supplemental Figure 4: Co-treatment with vorinostat and cisplatin synergistically induces apoptosis in breast cancer cells. **A**. HCC1937 and MDA-MB-231 cells were treated with cisplatin for 48 hours. The % annexin V-positive, apoptotic cells was determined by flow cytometry. Columns, mean of three experiments; Bars, SEM. **B**. HCC1937, MDA-MB-231 and SUM159PT cells were treated with cisplatin and vorinostat (VS) for 48 hours and the % apoptotic cells was determined by flow cytometry. Median dose effect and isobologram analyses were performed using Calcusyn. Combination index (CI) values less than 1.0 indicate a synergistic interaction of the two agents in the combination. **C**. MDA-MB-231, HCC1937 and SUM159PT cells were treated with the indicated concentrations of cisplatin and vorinostat (VS) for 48 hours and the % apoptotic cells were determined by flow cytometry. Median dose effect and isobologram analyses were performed using Calcusyn. Combination index (CI) values less than 1.0 indicate a synergistic interaction of the two agents in the combination. Figure shows the doses of each agent, the fraction of cells affected by the combination and the calculated CI value for each.
